# Activating Mn Sites by Ni Replacement in α-MnO_2_

**DOI:** 10.1021/acsmaterialsau.3c00051

**Published:** 2023-11-20

**Authors:** Sami M. Alharbi, Mohammed A. Alkhalifah, Benjamin Howchen, Athi N. A. Rahmah, Veronica Celorrio, David J. Fermin

**Affiliations:** † School of Chemistry, University of Bristol, Cantocks Close, Bristol BS8 1TS, U.K.; ‡ Department of Chemistry, College of Science, Qassim University, Buraydah 52571, Saudi Arabia; § Department of Chemistry, College of Science, King Faisal University, P.O. Box 380, Al-Ahsa, 31982, Saudi Arabia; ∥ Diamond Light Source Ltd., Diamond House, Harwell Campus, Didcot OX11 0DE, U.K.

**Keywords:** α-MnO_2_, Ni lattice replacement, oxygen reduction kinetics, electronic structure, active sites

## Abstract

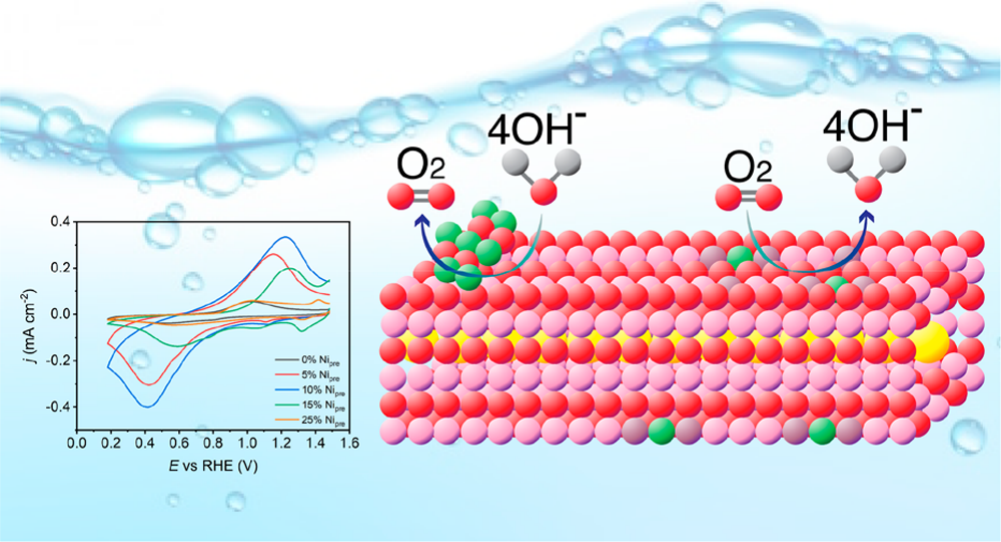

Transition metal oxides are characterized by an acute structure and composition dependent electrocatalytic activity toward the oxygen evolution (OER) and oxygen reduction (ORR) reactions. For instance, Mn containing oxides are among the most active ORR catalysts, while Ni based compounds tend to show high activity toward the OER in alkaline solutions. In this study, we show that incorporation of Ni into α-MnO_2_, by adding Ni precursor into the Mn-containing hydrothermal solution, can generate distinctive sites with different electronic configurations and contrasting electrocatalytic activity. The structure and composition of the Ni modified hollandite α-MnO_2_ phase were investigated by X-ray absorption spectroscopy (XAS), X-ray diffraction (XRD), transmission electron microscopy coupled to energy-dispersive X-ray spectroscopy (TEM-EDX), inductively coupled plasma–optical emission spectroscopy (ICP-OES), and X-ray photoelectron spectroscopy (XPS). Our analysis suggests that Mn replacement by Ni into the α-MnO_2_ lattice (site A) occurs up to approximately 5% of the total Mn content, while further increasing Ni content promotes the nucleation of separate Ni phases (site B). XAS and XRD show that the introduction of sites A and B have a negligible effect on the overall Mn oxidation state and bonding characteristics, while very subtle changes in the XPS spectra appear to suggest changes in the electronic configuration upon Ni incorporation into the α-MnO_2_ lattice. On the other hand, changes in the electronic structure promoted by site A have a significant impact in the pseudocapacitive responses obtained by cyclic voltammetry in KOH solution at pH 13, revealing the appearance of Mn 3d orbitals at the energy (potential) range relevant to the ORR. The evolution of Mn 3d upon Ni replacement significantly increases the catalytic activity of α-MnO_2_ toward the ORR. Interestingly, the formation of segregated Ni phases (site B) leads to a decrease in the ORR activity while increasing the OER rate.

## Introduction

The development of highly efficient oxygen electrocatalysts employing Earth-abundant elements is one of the key challenges in the development of scalable electrochemical energy conversion systems such as water electrolyzers, fuel cells, and metal air batteries.^1,2^ Complex transition metal oxides incorporating Co, Fe, Ir, Mn, Ni, and Ru have received a great deal of attention, with a diverse composition and coordination space that translates into a wide range of structural patterns, electronic properties, and electrocatalytic activity.^3−15^ Beyond aspects associated with binding energies of reactants and intermediates species, the whole electronic structure of these materials is also acutely sensitive to structure and composition, adding a significant level of complexity to the case of metallic phases.^11^

Mn-based oxides offer a fine example of how structure and coordination can affect their activity toward the oxygen reduction reaction.^16−22^ It has been reported that electrocatalytic activity toward the oxygen reduction reaction (ORR) for the various MnO_2_ phases follows the trend α- > β- > γ-MnO_2_.^13^ Other studies have shown that promoting oxygen vacancies in β-MnO_2_ can significantly increase their electrocatalytic performance.^23^ Another approach to enhance the activity of α-MnO_2_ is by introducing elements such as Ru, Co, Mg, Cu, Fe, Mo, and Ni-doped.^24−29^ The work by Selvakumar et al. proposed that deposition of Co and Ni onto α-MnO_2_ can lead to Mn sites with lower oxidation states, i.e., Mn(III) states, which are linked to a higher ORR activity.^30^ Indeed, several authors associate ORR activity with Mn(III) oxidation states.^2,26,31−33^ However, studies by Celorrio et al. on LaMnO_3_, SrMnO_3_, CaMnO_3_, and YMnO_3_ clearly demonstrated that there is no direct correlation between Mn oxidation state and ORR activity.^34^

In this work, we show for the first time that Ni incorporation into to the α-MnO_2_ lattice leads to activation of Mn sites (site A) toward the ORR, as opposed to Ni sites nucleated at the surface of the oxide (site B). Systematic structural and composition analysis based on X-ray absorption spectroscopy (XAS), X-ray diffraction (XRD), transmission electron microscopy coupled to energy-dispersive X-ray spectroscopy (TEM-EDX), inductively coupled plasma–optical emission spectroscopy (ICP-OES), and X-ray photoelectron spectroscopy (XPS) shows little differentiation between both types of sites in α-MnO_2_. On the other hand, clear contrasts can be seen in the pseudocapacitive electrochemical fingerprints measured in alkaline solutions. Site A, primarily formed under a low Ni content, promotes Mn 3d orbitals at energies (potentials) relevant to the ORR, leading to an increase in ORR kinetics. By contrast, site B is dominated by Ni 3d orbitals, which catalyzes the OER kinetics.

## Results and Discussion

High aspect ratio α-MnO_2_ nanostructures were prepared by an established hydrothermal method (details of experimental and synthesis methods are included in the Methods section),^30^ while Ni modified α-MnO_2_ nanostructures were prepared by adjusting the ratio of Ni:Mn introducing Ni(NO_3_)_2_·6H_2_O in the precursor solution. It is important to differentiate the Ni content in the precursor solution (experimental variable) denoted as Ni_pre_, from the mean Ni content (Ni_tot_) estimated by techniques such as ICP-OES and EDX, and from the surface Ni ratio (Ni_surf_) obtained from XPS. The Ni content in all cases is defined as the molar ratio of Ni/(Mn + Ni). We will be using this notation throughout the whole paper. The precursor solution was heated in a Teflon-lined stainless-steel autoclave at 140 °C for 12 h. The product was washed with distilled water, filtered, and then dried in the air.

**Figure 1:**
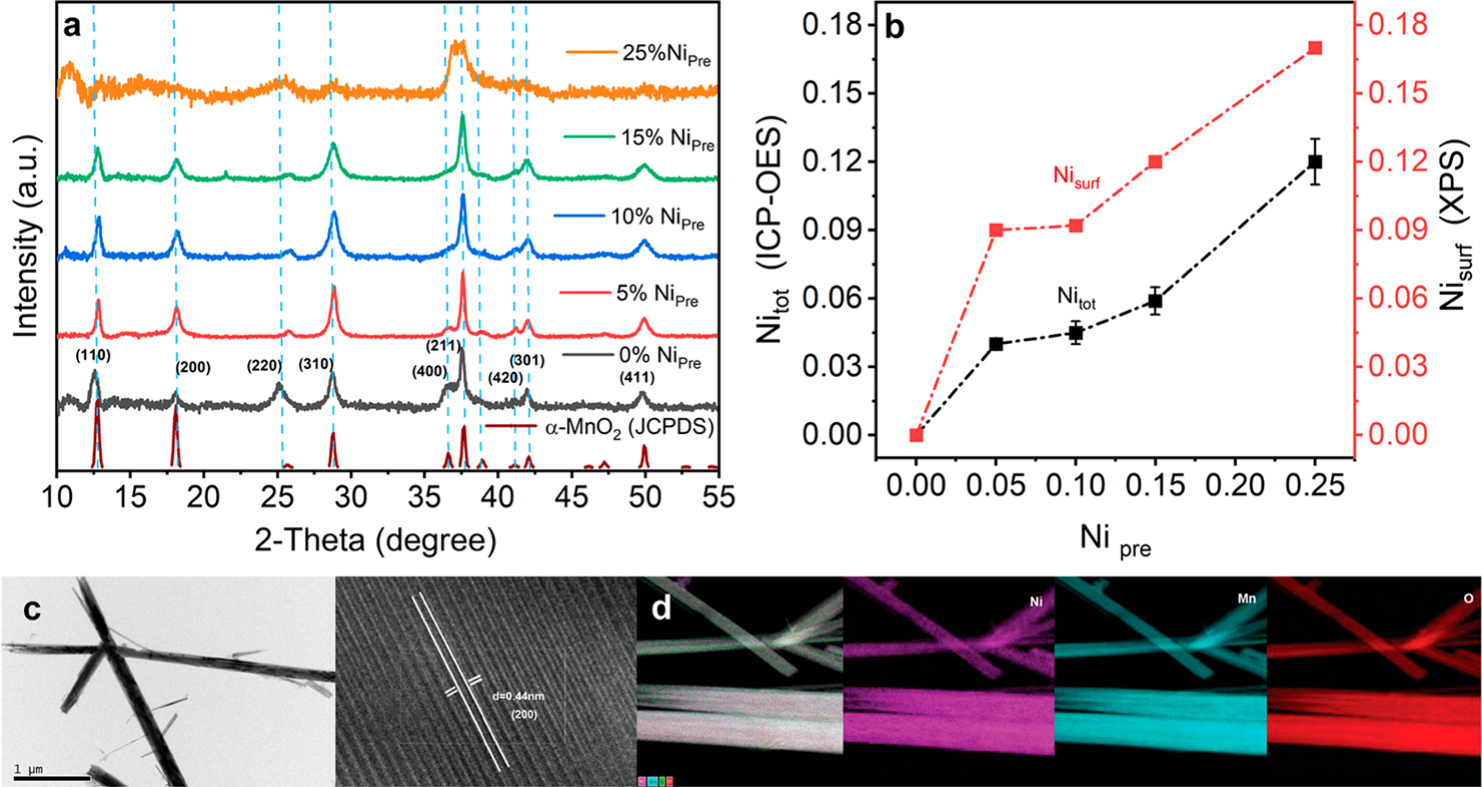
Structure and composition analysis of α-MnO_2_ with various Ni content: (a) XRD patterns of the materials obtained from precursors containing various Ni/(Ni + Mn) molar ratios (Ni_pre_). (b) Correlations between Ni_pre_ and the Ni–Mn molar ratio of the nanostructures estimated by ICP-OES (Ni_tot_) and the surface molar ration (Ni_surf_) obtained by XPS analysis. (c) Characteristic TEM images of 15% Ni_pre_ illustrating the high aspect ratio of the hollandite phase and lattice fringes, with 0.44 ± 0.2 nm d-spacing, associated with the (002) planes; scale bar corresponds to 1 μm. (d) TEM-EDX images showing distribution of Ni, Mn, and O across the 15% Nipre sample.

XRD patterns of the oxide with different Ni_pre_ content varying from 0 (α-MnO_2_) to 25% are displayed in Figure 1a. Materials prepared with up to 15% Ni_pre_ show clear diffraction peaks corresponding to (110), (200), (220), (310), (400), (211), (420), (301), and (411) of hollandite α-MnO_2_ (JCPDS file 00-44-0141) and a space group of 14/*m*. The significant broadening of the XRD features in 25% Ni_pre_ suggests that the crystalline domain size of the oxide is decreased with respect to the lower Ni content. Figure 1b reveals interesting correlations between the Ni_pre_ composition and the total Ni content measured in the nanostructures (Ni_tot_) and at the surface (Ni_surf_) as probed by ICP-OES and XPS. Ni_tot_ changes only between 4.5% and 6% upon increasing Ni_pre_ up to 15%. XPS composition analysis shows a similar trend, although the Ni_surf_ values are slightly higher than Ni_tot_ values. In principle, this could be rationalized as Ni concentration grading from the bulk toward the surface. However, α-MnO_2_ is characterized by its high aspect ratio, as shown in Figure 1c; thus, the interplay of surface density of Mn sites and XPS penetration depth is far from trivial. Increasing Ni_pre_ above 15% leads to a jump in Ni content in both sets of measurements.

Figure 1c show characteristic transmission electron microscopy images of the 15% Ni_pre_, illustrating the high aspect ratio characteristic of α-MnO_2_ as well as lattice fringes with 0.44 nm *d*-spacing associated with the (200) planes. Figure S1 of the Supporting Information show additional TEM images of materials obtained across the Ni_pre_ content investigated. All materials exhibit the characteristic high aspect ratio of the hollandite phase along with the lattice fringes associated with the (002) planes, which is also consistent with the phase purity of the material observed by XRD up to 15% Ni_pre_. In the case of 25% Ni_pre_, Figure S1 shows that the dimensions of the α-MnO_2_ crystals are reduced along with the appearance of particles with different morphology. TEM-EDX images in Figure S2 show that the featureless particles are primarily composed of Ni. The apparent decrease in α-MnO_2_ crystal size in Ni_pre_ 25% is consistent with the broadening of the XRD features observed in Figure 1a, while the absence of clear diffraction features associated with Ni oxide phases suggests that the segregated material is amorphous. On the other hand, Figure S2 also shows that Ni is primarily found along the α-MnO_2_ lattice up to Ni_pre_ 15%. Tables S1 and S2 show that the compositions extracted from EDX and ICP-OES are consistent, including the content of K^+^ ions which are often linked to charge stabilization in the inside the [2 × 2] channel.^35^

**Figure 2:**
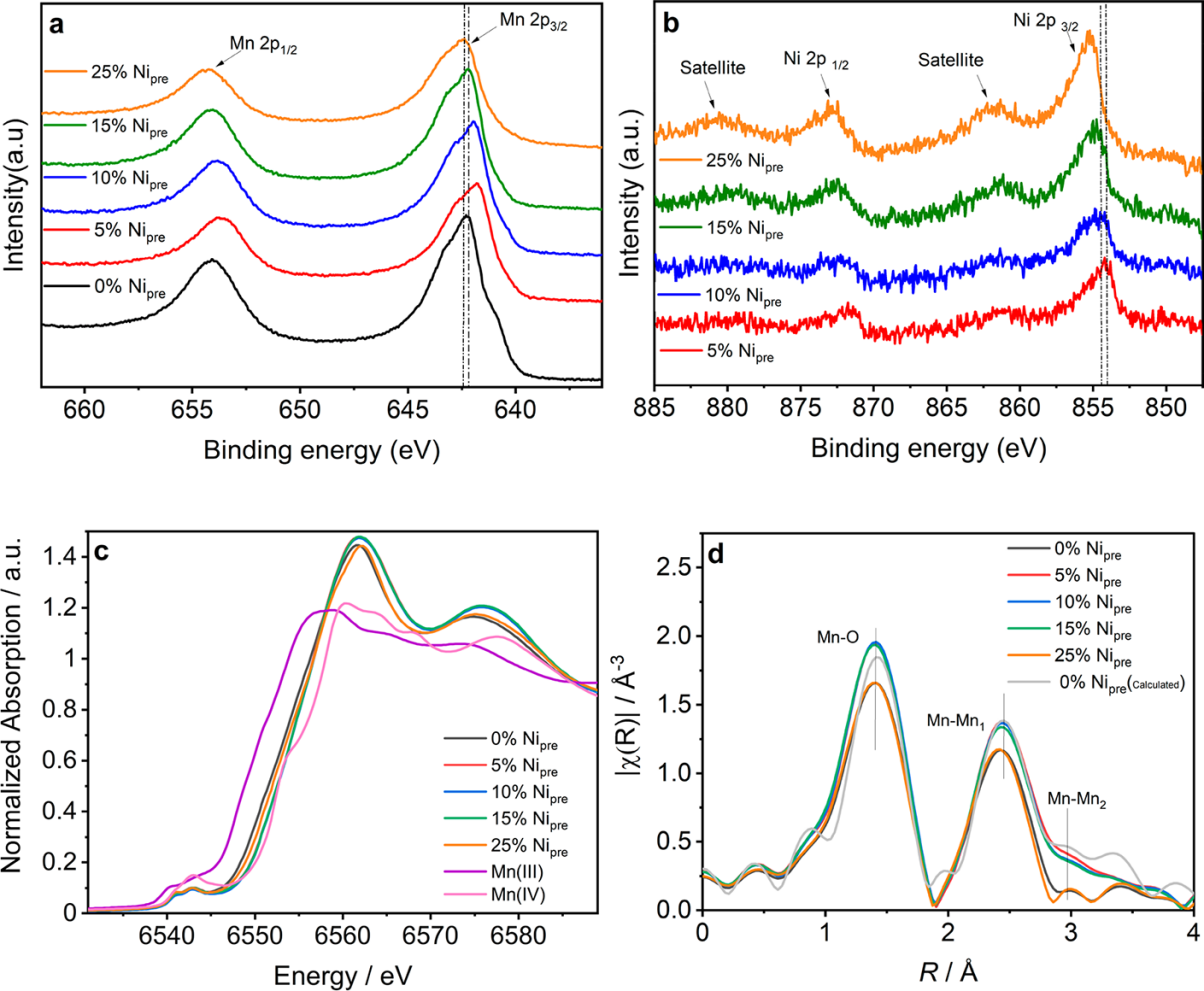
Surface composition and bonding analysis: (a) XPS spectra of Mn 2p showing that the oxidation state of Mn appears to be affected little by the introduction of Ni. (b) XPS spectra of Ni 2p, in which the Ni 2p_3/2_ slightly shifts to higher binding energies as the Ni_pre_ content increases. (c) Normalized XAS spectra contrasting the edge of Mn(II) and Mn (IV) standard with those of α-MnO_2_ obtained with different Ni_pre_. (d) FT of the k^3^-weighted EXAFS spectra showing the first and second coordination shell for the various Ni_pre_ samples.

X-ray photoemission spectra (XPS) of Mn 2p and Ni 2p orbitals are shown for the different formulated electrocatalysts, shown in Figure 2a and b, respectively. Characteristic survey spectra of 5% and 25% Ni_pre_ are shown in Figure S3. The Mn 2p spectra exhibit a broad emission at 642.6 eV corresponding to Mn 2p_3/2_, and even broader and asymmetric emission at 654.3 eV which is linked to Mn 2p_1/2_. The broadening of the latter originates from the strong overlap of Mn^3+^ (641.9 eV) and Mn^4+^ (642.2 eV) sites at the oxide surface,^36−38^ which makes these contributions difficult to be accurately deconvoluted. However, it could be argued that the Mn 2p_1/2_ photoemission peak is slightly shifted toward lower binding energies (BEs) in the case of Ni_pre_ 5 and 10%, in comparison to Ni_pre_ 0 and 25%. On the other hand, there is a more systematic shift in the maximum of the Ni 2p_3/2_ photoemission line as the Ni content increases as shown in Figure 2b. These observations may suggest changes in the surface electronic configuration of Mn sites at a low Ni content, which effectively disappear once Ni nucleates separately from the α-MnO_2_ lattice.

Figure 2c and d show the Mn-edge of the X-ray absorption spectra (XAS) and the FT of the k^3^-weighted EXAFS spectra for the various materials, respectively. The position of the Mn K-edge does not show any systematic change with Ni content, indicating that there is no notable change in oxidation state on Mn. Figure 2d shows the comparison between the Fourier transforms (FTs) of the EXAFS data for the different compositions, in comparison with spectra of α-MnO_2_ (simulated from reference data for COD ID 1514116 downloaded from the Crystallography Open Database). It can be observed that the data collected for the synthesized samples strongly resemble that of the theoretical α-MnO_2_ structure. The amplitude reduction factor (S_0_^2^), bond length, Debye–Waller factor (σ^2^), and energy shift parameter (Δ*E*_0_) were refined. The best-fit parameters are summarized in Table S3, and data and fits are provided in Figure S4. The data confirm that there are no extended structural changes in the Mn coordination even at high Ni content, which is expected given their similar ionic radii. However, as demonstrated below, Ni replacement does introduce discrete changes in the structural/electronic configuration which cannot be detected in XAS spectra.

**Figure 3:**
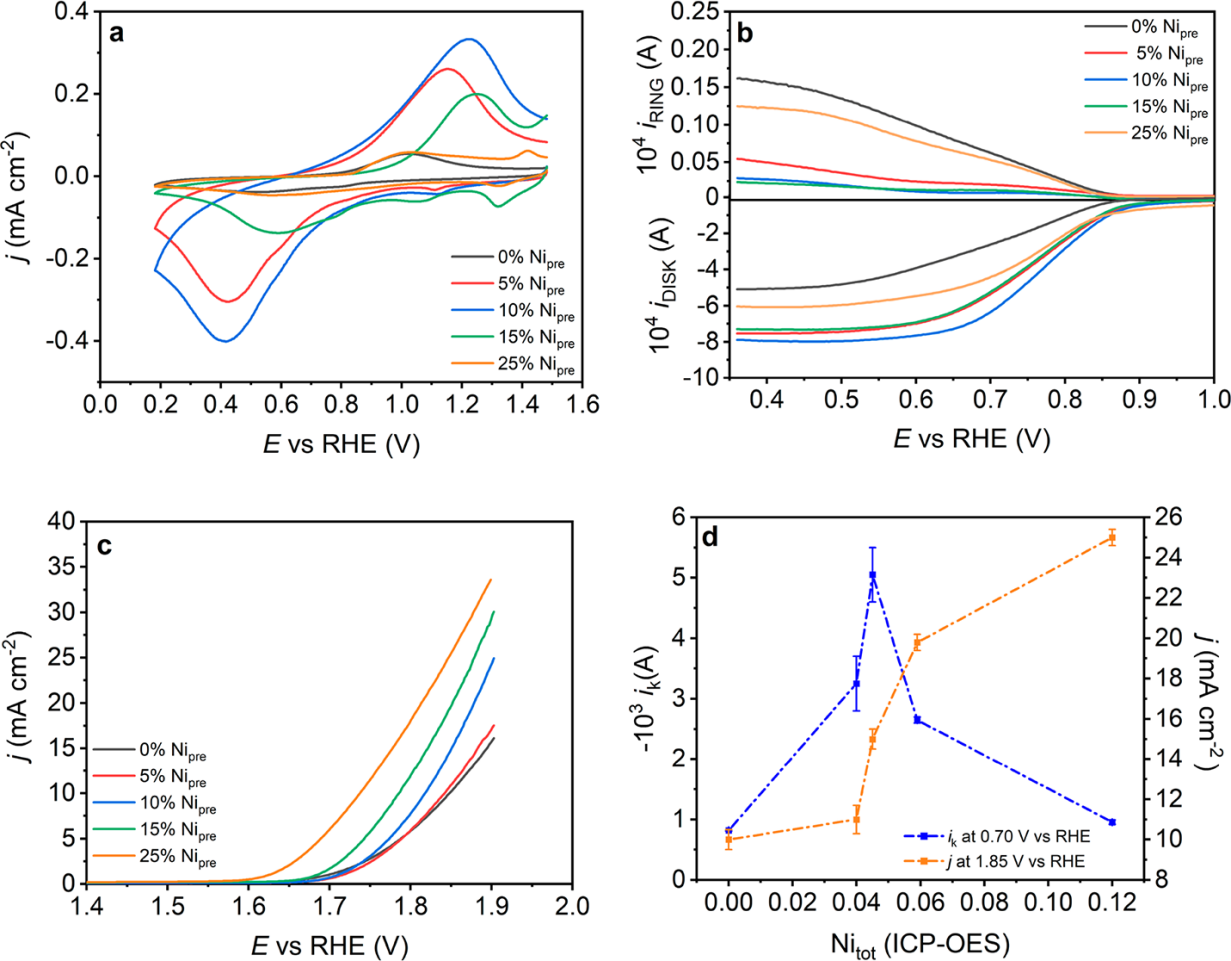
Electrochemical responses and electrocatalytic activity: (a) Pseudocapacitance responses of Ni doped MnO_2_ catalyst layers at a glassy carbon (GC) electrode in Ar-saturated 0.1 M KOH at scan rate 10 mV s^–1^. (b) Rotating ring-disk electrode measurements at 1600 rpm for the various catalyst compositions in O_2_-saturated 0.1 M KOH with a sweep rate of 10 mV s^–1^, with the Pt ring electrode held constant at 1.2 vs RHE. (c) Linear sweep voltammetry of α-MnO_2_ obtained with different Ni_pre_ content in 0.1 M KOH with a scan rate of 10 mV s^–1^ and 1600 rpm. (d) Kinetically limited current (*i*_k_) for the ORR at 0.70 V vs RHE and current density (normalized by geometric area) for the OER at 1.85 V vs RHE as a function of the actual Ni content in the nanostructure (Ni_tot_).

Figure 3a contrasts cyclic voltammograms of α-MnO_2_, obtained with various Ni_pre_ content, supported on a mesoporous carbon layer with a 398 μg cm^–2^ oxide loading, 50 μg cm^–2^ Vulcan, and 50 μg cm^–2^ Nafion (see Methods section) in argon-saturated 0.1 M KOH solutions at 10 mV s^–1^. α-MnO_2_ is characterized by a broad pseudocapacitive reduction peak centered at potentials close to 0.6 V vs RHE, and a sharper oxidation peak in the reversed scan at 1.0 V vs RHE. Studies in the literature associate these signals with Mn^4+^/Mn^2+^ redox transition.^33,39−42^ We correlate these signals with the density and distribution of Mn 3d states across the potential (energy) region relevant to oxygen electrocatalysis.^6,33^ Interestingly, 5 and 10% Ni_pre_ are characterized by strong pseudocapacitive responses, leading to a broad cathodic current with a peak centered at 0.4 V and an anodic peak at about 1.3 V in the reverse scan. Smaller features can be observed between 1 and 1.2 V in the forward (negative) scan, but the main observation is the substantial increase in the density of Mn 3d states upon introducing Ni into the lattice. This observation reveals the emergence of a new local electronic configuration, which we will refer to as site A, in which Mn displays a higher local density of 3d states at a potential relevant to the ORR. A dampening of these responses is observed in 15% and 25% Ni_pre_, along with the emergence of a different redox transition between 1.2 and 1.4 V vs RHE. This new redox transition is associated with changes in the oxidation state of Ni,^30,34,35,40,43,44^ which coincides with the onset of Ni phase segregation as discussed previously. These observations suggest that Ni segregation generates a different site (Site B) which has a negligible effect on the electronic properties α-MnO_2_.

Figure 3b illustrates the ORR current responses on a rotating-ring disk electrode (RRDE) at 1600 rpm in an O_2_-staurated 0.1 M KOH solution, with the Pt ring potential fixed at 1.2 V vs RHE (generation-collection mode). The results clearly show that the introduction of Ni decreases the onset potential for the ORR and increases the overall current with respect to α-MnO_2_. The Pt ring current decreases as Ni_pre_ increases to 15%, which is associated with the rate of HO_2_^–^ generated at the disk electrode. Interestingly, the rate of HO_2_^–^ generation increases in the case of 25% Ni_pre_. Figure 3c contrasts linear sweep voltammetry (LSV) in the OER potential range, showing a systematic shift of the onset potential of the Ni in the Ni content increases. The IR drop in our electrode configuration is independent of the catalyst composition with an average of 10 ± 2 Ω. Figure S6a shows that IR compensation has a minor effect on the current–potential dependence. Tafel plots (Figure S6b) also show that the effective transfer coefficient is little dependent on the Ni content. Stability studies under OER conditions, as those reported in other works,^45,46^ are outside of the scope of this work, which primarily focus on the contrasting nature of the Ni generated active sites. Potential induced surface reconstruction is another important aspect that is not explicitly considered in our analysis, which would require carefully conducted experiments at single crystal electrodes.

Figure 3d shows the dependence of the kinetically limiting current (*i*_k_) at 0.70 V vs RHE, as estimated from Koutecky–Levich analysis, on the total Ni molar ratio in the nanostructures (Ni_tot_). Details of this analysis are shown in the Methods section and are exemplified in Figure S5. We can see a sharp increase in the *i*_k_ value, indicating an increase in ORR activity up to Ni_tot_ of 4.5%, followed by a decrease at higher content. Analysis of the effective number of electrons in the ORR process (Table S4) based on the generation-collection RRDE configuration (see Methods) shows that α-MnO_2_ primarily reduces oxygen through the two-electron process, generating HO_2_^–^ under alkaline conditions. Introducing 5–10% Ni to the precursor solution, which leads to primarily site A, swiftly changes the mechanism to the four-electron process. Further increasing the Ni content in the precursor, promoting site B instead, switches the reaction pathway back to the two-electron process. This behavior indicates that the ORR kinetics (*i*_k_) and pathways are dictated by the emergence of site A. On the other hand, the OER current measured at 1.85 V vs RHE exhibits a monotonic increase with increasing Ni content.

Our analysis provides unambiguous evidence that Ni insertion into the α-MnO_2_ lattice (site A) leads to a distortion of the electronic structure which manifests itself by an increase of Mn 3d states at potentials relevant to the ORR. This can be clearly seen by the emergence of the pseudocapacitive responses for Ni_tot_ in the range of 5%. Although we have not been able to determine the changes in the electronic configuration associated with site A, we do not see this as just changes in the local oxidation state. As demonstrated in our previous works, Mn oxides with a nominal +3 oxidation state such us YMnO_3_ are rather inactive,^34^ while compounds such CaMnO_3_ (Mn^4+^) are active toward the ORR.^47^ Attempting to increase the Ni content by increasing Ni_pre_ leads to the formation of surface segregated Ni phases (site B) which are less active toward the ORR. It is interesting to contrast these observations with our previous analysis of LaMn_*x*_Ni_1–*x*_O_3_,^6^ where we see a systematic decrease in ORR activity and increase in OER activity with increasing Ni content. In the case of the mixed perovskite, replacing Ni by Mn leads to minimal structural changes, while electronic interactions between Ni and Mn are also relatively weak.

## Conclusions

This study reveals that insertion of Ni into α-MnO_2_ can lead to an increase in the activity toward the ORR only if the electronic structure of the oxide is distorted such that Mn 3d states are generated at the relevant potential (energy) scale. By introducing Ni into the hydrothermal precursor solution, we were able to incorporate Ni up to approximately 5% of the total cation content in the oxide lattice. Further increase of the Ni precursors leads to the nucleation of Ni phases at the surface of α-MnO_2_, which are less active toward the ORR. The emergence of active Mn sites (site A), promoted by the insertion Ni, is observed by the evolution of pseudocapacitive responses between 0.2 and 1.2 V vs RHE. On the other hand, the OER kinetics monotonically increase with increasing overall Ni content in the catalysts. This is an important observation, demonstrating that ORR is extremely sensitive to structure and coordination of Mn sites, while OER is primarily determined by number density of Ni sites. Further studies are required to identify the exact nature of the ORR active sites promoted by Ni replacement, which could not be clearly identified from XAS, XPS, or TEM analysis. In any case, these studies further confirm the presence of pseudocapacitive responses associated with d-states as an effective descriptor of electrocatalytic activity in these complex materials. In more molecular terms, the oxidation state is not the determining factor as such but rather changes in oxidation states which arises from populating/depopulating d-states in the potential range relevant for catalysis.

## Methods

### Materials Synthesis

High purity KMnO_4_, MnCl_2_·4H_2_O, Ni(NO_3_)_2_·6H_2_O, Vulcan XC 72R, and Nafion were purchased from Sigma-Aldrich. α-MnO_2_ was synthesized by a hydrothermal method following reports published elsewhere.^19,23^ Amounts of 0.5 g of KMnO_4_ and 0.2 g of MnCl_2_·4H_2_O were added to 15 mL of Milli-Q water under magnetic stirring for 10 min. The solution was transferred to a Teflon-lined stainless-steel autoclave, which was sealed and heated at 140 C for 12 h. The Teflon-lined stainless-steel autoclave was naturally cooled down to the room temperature, and then the product was centrifuged three times with Milli-Q water. After that, the precipitate was dried in a vacuum oven at 80 C for 12 h. Ni-modified α-MnO_2_ was synthesized through the same hydrothermal method, adjusting the Ni/(Ni + Mn) molar ratio in the precursor (Ni_pre_) by adding Ni(NO_3_)_2_·6H_2_O.

### Electrocatalyst Catalyst Layer

The first step in the preparation of the electrocatalyst ink involved mixing 16 mg of Vulcan XC 72R, 1 mL of Nafion solution as a perfluorinated resin binder, and 15 mL of deionized water. The mixture was dispersed in an ultrasound bath for 40 min. Then 2.5 mg of the oxide electrocatalyst was mixed with 500 μL of the ink and sonicated for 30 min to ensure a homogeneous suspension. Prior to the deposition of the catalysts, the rotating ring-disk electrode (RRDE) was polished with alumina powder and Milli-Q water. Immediately after sonication of the precursor solution, 10 μL of the ink was carefully drop casted onto the freshly polished glassy carbon disk, minimizing contamination of the Pt ring. The catalyst layer was left to dry for 5 min with a hot air gun. The catalyst loading at the electrode was 398 μg cm^–2^.

### Material Characterization and Electrochemical Studies

X-ray diffraction (XRD) patterns were recorded utilizing a Bruker AXS D8 Advance diffractometer with a θ–θ configuration, utilizing Cu Kα radiation (λ = 0.154 nm). The diffraction patterns were recorded at 25 °C with a step size of 0.02° and a time per step of 2 s over an angular range of 10–55°. A JEOL JEM 2010 transmission electron microscope fitted with a Gatan Orius digital camera was used in these studies, coupled to an energy-dispersive X-ray spectroscopy (EDX) detector. Samples were prepared for TEM analysis by dispersing the powder in ethanol utilizing ultrasonication and pipetting 1000 μL drops of ethanol dispersed onto 3 mm diameter copper grids.

Inductively coupled plasma atomic emission spectroscopy (ICP-OES) was conducted on an Agilent 710 simultaneous spectrometer. The sample preparation was 5 mg of each sample dissolved in 1 cm^–3^ nitric acid (1 wt %) which was diluted to reach a final volume of 10 cm^–3^ by adding Milli-Q water. The standard solutions contain Mn, K, and Ni, respectively, all dissolved in a 1 wt % nitric acid (supra pure). The wavelengths used for quantification of Mn were 257.610, 259.372, 260.568, 294.921 nm, while 216.555, 221.648, 230.299, and 231.604 nm lines were used for Ni quantification.

XAS spectra were acquired at the B18 beamline of the Diamond Light Source, featuring a Canberra 35-element monolithic planar Ge pixel array detector in fluorescence mode at the Mn K-edge (6539 eV). The samples were made into pellets (1.32 cm^2^ pellet area) by combining the ground sample with cellulose (80 mg) to obtain a homogeneous mixture, which was then compressed (5 tonnes) by using pellet press. The Mn K-edge range was established by using Mn foil. Data analysis was performed with Athena and Artemis software.

X-ray photoelectron spectroscopy (XPS) analysis was conducted in a NanoESCA II instrument at ambient temperature and under ultrahigh vacuum (4 × 10^–11^ mbar). The photoemission spectra of C 1s, O 1s, Mn 2p, and Ni 2p were recorded at room temperature. Spectra were recorded using a step size of 0.1 eV, a collection time of 0.5 s, and a pass energy of 20 eV. Binding energies (BEs) were calibrated using the C 1s peak (284.6 eV) as reference. Surface composition of the materials was measured from high-resolution Ni 2p and Mn 2p spectra.

Electrochemical measurements were conducted in a three-electrode cell by using a rotating ring-disk electrode (RRDE) attached to an ALS rotation control system and linked to a CompactStat bipotentiostat (Ivium). The RRDE electrode was made up of a catalyst casted onto a glassy carbon disk 4 mm diameter (0.126 cm^2^ surface area) surrounded by a Pt ring 7 mm inner diameter, both of which served as the working electrode. Hg/HgO was utilized as the reference electrode, and a carbon rod was used as the counter electrode. In this work, all of the potentials have been converted in reference to an RHE scale. Pseudocapacitive responses were measured by cyclic voltammetry (CV) in argon-saturated 0.1 M KOH electrolyte at 10 mV scan rates. ORR kinetics were obtained from linear sweep voltammetry studies at 10 mV s^–1^ in O_2_ saturated 0.1 M KOH solution employing RRDE electrodes. The effective number of electrons transferred (*n*) can be calculated from the currents at the disk (*i*_D_) and ring (*i*_R_) electrodes based on
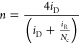
1where *N*_c_ is the collection efficiency. The Pt ring electrode is held constant at 1.2 V vs RHE while the disk electrode is swept across the potential range. The kinetically limited current (*i*_k_) for the ORR was calculated from the Koutecky–Levich expression
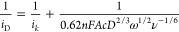
2where *c*, *D*, ω, and ν correspond the bulk oxygen concentration (1.2 × 10^–6^ mol cm^–3^), the oxygen diffusion coefficient (1.9 × 10^–5^ cm^2^ s^–1^), the angular rate, and the kinematic viscosity of water (0.01 cm^2^ s^–1^), respectively.

## Data Availability

Data are available at the University of Bristol data repository, data.bris, at https://doi.org/10.5523/bris.3q1p0lek67uxw2v45atcsjo34p.

## Supporting Information Available

High resolution TEM images of α-MnO_2_ with various Ni content; TEM-EDX images of α-MnO_2_ with various Ni content; XPS survey spectra of 5% and 25% Ni_pre_; k2-weighted EXAFS signals in k-space and magnitude of FT signal of the k2-weighted EXAFS spectra of α-MnO_2_ with various Ni content; Koutecky–Levich plots of the ORR reaction at 0.70 V vs RHE at α-MnO_2_ electrocatalysts with various Ni content; LSV curves in the OER region including and excluding IR compensation as well as Tafel plots for the various Ni modified α-MnO_2_; elemental composition of α-MnO_2_ with various Ni content as obtained from ICP-OES; elemental composition of α-MnO_2_ with various Ni content as obtained from EDX; structural parameters obtained from EXAFS analysis; effective number of electrons (*n*) and kinetically limited current (*i*_k_) estimated at 0.70 V vs RHE obtained from the rotating ring-disk electrode responses at various angular rotation rates (PDF)

## References

[ref1] Winter M.; Brodd R. J. What Are Batteries, Fuel Cells, and Supercapacitors?. Chem. Rev. 2004, 104 (10), 4245–4270. 10.1021/cr020730k. 15669155

[ref2] Cheng F.; Chen J. Metal-Air Batteries: from Oxygen Reduction Electrochemistry to Cathode Catalysts. Chem. Soc. Rev. 2012, 41 (6), 2172–2192. 10.1039/c1cs15228a. 22254234

[ref3] Xiong Y.; Yang Y.; Feng X.; DiSalvo F. J.; Abruña H. D. A Strategy for Increasing the Efficiency of the Oxygen Reduction Reaction in Mn-Doped Cobalt Ferrites. J. Am. Chem. Soc. 2019, 141 (10), 4412–4421. 10.1021/jacs.8b13296. 30789717

[ref4] Bonnefont A.; Ryabova A. S.; Schott T.; Kéranguéven G.; Istomin S. Y.; Antipov E. V.; Savinova E. R. Challenges in the Understanding Oxygen Reduction Electrocatalysis on Transition Metal Oxides. Curr. Opin. Electrochem. 2019, 14, 23–31. 10.1016/j.coelec.2018.09.010.

[ref5] Celorrio V.; Tiwari D.; Fermin D. J. Composition-Dependent Reactivity of Ba_0. 5_Sr_0. 5_Co_x_Fe_1–x_O_3−δ_ toward the Oxygen Reduction Reaction. J. Phys. Chem. C 2016, 120 (39), 22291–22297. 10.1021/acs.jpcc.6b04781.

[ref6] Alkhalifah M. A.; Howchen B.; Staddon J.; Celorrio V.; Tiwari D.; Fermin D. J. Correlating Orbital Composition and Activity of LaMn_x_Ni_1–x_O_3_ Nanostructures toward Oxygen Electrocatalysis. J. Am. Chem. Soc. 2022, 144 (10), 4439–4447. 10.1021/jacs.1c11757. 35254811 PMC9097476

[ref7] Stoerzinger K. A.; Risch M.; Han B.; Shao-Horn Y. Recent Insights into Manganese Oxides in Catalyzing Oxygen Reduction Kinetics. ACS Catal. 2015, 5 (10), 6021–6031. 10.1021/acscatal.5b01444.

[ref8] Han M.; Gómez-Recio I.; Martín D. G.; Ortiz Peña N.; Ruiz-González M. L.; Selmane M.; González-Calbet J. M.; Ersen O.; Zitolo A.; Lassalle-Kaiser B.; et al. Tuning of Oxygen Electrocatalysis in Perovskite Oxide Nanoparticles by the Cationic Composition. ACS Catal. 2023, 13 (8), 5733–5743. 10.1021/acscatal.3c00461.

[ref9] Weng Z.; Huang H.; Li X.; Zhang Y.; Shao R.; Yi Y.; Lu Y.; Zeng X.; Zou J.; Chen L.; et al. Coordination Tailoring of Epitaxial Perovskite-Derived Iron Oxide Films for Efficient Water Oxidation Electrocatalysis. ACS Catal. 2023, 13 (4), 2751–2760. 10.1021/acscatal.2c05147.

[ref10] Bacirhonde P. M.; Mohamed A. Y.; Han B.; Cho D. Y.; Devendra S.; Choi J. W.; Lim C. R.; Afranie E. O.; Baik K. H.; Kang K.; et al. Ruthenium Engineered A_2_B_2_O_6_-Hybrid Columbite Ferrite for Bifunctional pH-Universal Water Splitting. Adv. Energy Mater. 2023, 13, 2300174 10.1002/aenm.202300174.

[ref11] Chong L.; Gao G.; Wen J.; Li H.; Xu H.; Green Z.; Sugar J. D.; Kropf A. J.; Xu W.; Lin X.-M.; et al. La-and Mn-Doped Cobalt Spinel Oxygen Evolution Catalyst for Proton Exchange Membrane Electrolysis. Science 2023, 380 (6645), 609–616. 10.1126/science.ade1499. 37167381

[ref12] Suntivich J.; May K. J.; Gasteiger H. A.; Goodenough J. B.; Shao-Horn Y. A Perovskite Oxide Optimized for Oxygen Evolution Catalysis from Molecular Orbital Principles. Science 2011, 334 (6061), 1383–1385. 10.1126/science.1212858. 22033519

[ref13] Suntivich J.; Gasteiger H. A.; Yabuuchi N.; Nakanishi H.; Goodenough J. B.; Shao-Horn Y. Design Principles for Oxygen-Reduction Activity on Perovskite Oxide Catalysts for Fuel Cells and Metal–Air Batteries. Nat. Chem. 2011, 3 (7), 546–550. 10.1038/nchem.1069. 21697876

[ref14] Celorrio V.; Tiwari D.; Calvillo L.; Leach A.; Huang H.; Granozzi G.; Alonso J. A.; Aguadero A.; Pinacca R. M.; Russell A. E.; et al. Electrocatalytic Site Activity Enhancement via Orbital Overlap in A_2_MnRuO_7_ (A = Dy^3+^, Ho^3+^, and Er^3+^) Pyrochlore Nanostructures. ACS Appl. Energy Mater. 2021, 4 (1), 176–185. 10.1021/acsaem.0c02060.

[ref15] Celorrio V.; Dann E.; Calvillo L.; Morgan D. J.; Hall S. R.; Fermin D. J. Oxygen Reduction at Carbon-Supported Lanthanides: The Role of the B-Site. ChemElectroChem. 2016, 3 (2), 283–291. 10.1002/celc.201500440.

[ref16] Reier T.; Nong H. N.; Teschner D.; Schlögl R.; Strasser P.; et al. Electrocatalytic Oxygen Evolution Reaction in Acidic Environments–Reaction Mechanisms and Catalysts. Adv. Energy Mater. 2017, 7 (1), 1601275 10.1002/aenm.201601275.

[ref17] Xiao W.; Wang D.; Lou X. W. Shape-Controlled Synthesis of MnO_2_ Nanostructures with Enhanced Electrocatalytic Activity for Oxygen Reduction. J. Phys. Chem. C 2010, 114 (3), 1694–1700. 10.1021/jp909386d.

[ref18] Cheng F.; Su Y.; Liang J.; Tao Z.; Chen J. MnO2-Based Nanostructures as Catalysts for Electrochemical Oxygen Reduction in Alkaline Media. Chem. Mater. 2010, 22 (3), 898–905. 10.1021/cm901698s.

[ref19] Lima F. H. B.; Calegaro M. L.; Ticianelli E. A. Electrocatalytic Activity of Manganese Oxides Prepared by Thermal Decomposition for Oxygen Reduction. Electrochim. Acta 2007, 52 (11), 3732–3738. 10.1016/j.electacta.2006.10.047.

[ref20] Morita M.; Iwakura C.; Tamura H. The Anodic Characteristics of Massive Manganese Oxide Electrode. Electrochim. Acta 1979, 24 (4), 357–362. 10.1016/0013-4686(79)87019-X.

[ref21] Celorrio V.; Calvillo L.; van den Bosch C. A. M.; Granozzi G.; Aguadero A.; Russell A. E.; Fermin D. J. Mean Intrinsic Activity of Single Mn Sites at LaMnO_3_ Nanoparticles Towards the Oxygen Reduction Reaction. ChemElectroChem. 2018, 5 (20), 3044–3051. 10.1002/celc.201800729.

[ref22] Gobaille-Shaw G. P. A.; Celorrio V.; Calvillo L.; Morris L. J.; Granozzi G.; Fermín D. J. Effect of Ba Content on the Activity of La_1-x_Ba_x_MnO_3_ towards the Oxygen Reduction Reaction. ChemElectroChem. 2018, 5 (14), 1922–1927. 10.1002/celc.201800052. 30263882 PMC6146913

[ref23] Cheng F.; Zhang T.; Zhang Y.; Du J.; Han X.; Chen J. Enhancing Electrocatalytic Oxygen Reduction on MnO_2_ with Vacancies. Angew. Chem. 2013, 125 (9), 2534–2537. 10.1002/ange.201208582. 23355314

[ref24] Meng Y.; Song W.; Huang H.; Ren Z.; Chen S.-Y.; Suib S. L. Structure–Property Relationship of Bifunctional MnO_2_ Nanostructures: Highly Efficient, Ultra-Stable Electrochemical Water Oxidation and Oxygen Reduction Reaction Catalysts Identified in Alkaline Media. J. Am. Chem. Soc. 2014, 136 (32), 11452–11464. 10.1021/ja505186m. 25058174

[ref25] Lehtimäki M.; Hoffmannová H.; Boytsova O.; Bastl Z.; Busch M.; Halck N. B.; Rossmeisl J.; Krtil P. Targeted Design of α-MnO_2_ Based Catalysts for Oxygen Reduction. Electrochim. Acta 2016, 191, 452–461. 10.1016/j.electacta.2016.01.070.

[ref26] Davis D. J.; Lambert T. N.; Vigil J. A.; Rodriguez M. A.; Brumbach M. T.; Coker E. N.; Limmer S. J. Role of Cu-Ion Doping in Cu-α-MnO_2_ Nanowire Electrocatalysts for the Oxygen Reduction Reaction. J. Phys. Chem. C 2014, 118 (31), 17342–17350. 10.1021/jp5039865.

[ref27] Lambert T. N.; Vigil J. A.; White S. E.; Delker C. J.; Davis D. J.; Kelly M.; Brumbach M. T.; Rodriguez M. A.; Swartzentruber B. S. Understanding the Effects of Cationic Dopants on α-MnO_2_ Oxygen Reduction Reaction Electrocatalysis. J. Phys. Chem. C 2017, 121 (5), 2789–2797. 10.1021/acs.jpcc.6b11252.

[ref28] Pargoletti E.; Cappelletti G.; Minguzzi A.; Rondinini S.; Leoni M.; Marelli M.; Vertova A. High-Performance of Bare and Ti-Doped α-MnO_2_ Nanoparticles in Catalyzing the Oxygen Reduction Reaction. J. Power Sources 2016, 325, 116–128. 10.1016/j.jpowsour.2016.06.020.

[ref29] Roche I.; Chaînet E.; Chatenet M.; Vondrák J. Carbon-Supported Manganese Oxide Nanoparticles as Electrocatalysts for the Oxygen Reduction Reaction (ORR) in Alkaline Medium: Physical Characterizations and ORR Mechanism. J. Phys. Chem. C 2007, 111 (3), 1434–1443. 10.1021/jp0647986.

[ref30] Selvakumar K.; Duraisamy V.; Venkateshwaran S.; Arumugam N.; Almansour A. I.; Wang Y.; Xiaoteng Liu T.; Murugesan Senthil Kumar S. Development of α-MnO_2_ Nanowire with Ni-and (Ni, Co)-Cation Doping as an Efficient Bifunctional Oxygen Evolution and Oxygen Reduction Reaction Catalyst. ChemElectroChem. 2022, 9 (2), e202101303 10.1002/celc.202101303.

[ref31] Stoerzinger K. A.; Lu W.; Li C.; Ariando; Venkatesan T.; Shao-Horn Y. Highly Active Epitaxial La_(1–x)_Sr_x_MnO_3_ Surfaces for the Oxygen Reduction Reaction: Role of Charge Transfer. J. Phys. Chem. Lett. 2015, 6 (8), 1435–1440. 10.1021/acs.jpclett.5b00439. 26263148

[ref32] Ryabova A. S.; Napolskiy F. S.; Poux T.; Istomin S. Y.; Bonnefont A.; Antipin D. M.; Baranchikov A. Y.; Levin E. E.; Abakumov A. M.; Kéranguéven G.; et al. Rationalizing the Influence of the Mn (IV)/Mn (III) Red-O_x_Ttransition on the Electrocatalytic Activity of Manganese Oxides in the Oxygen Reduction Reaction. Electrochim. Acta 2016, 187, 161–172. 10.1016/j.electacta.2015.11.012.

[ref33] Celorrio V.; Leach A. S.; Huang H.; Hayama S.; Freeman A.; Inwood D. W.; Fermin D. J.; Russell A. E. Relationship between Mn Oxidation State Changes and Oxygen Reduction Activity in (La, Ca)MnO_3_ as Probed by In Situ XAS and XES. ACS Catal. 2021, 11 (11), 6431–6439. 10.1021/acscatal.1c00997.

[ref34] Celorrio V.; Calvillo L.; Granozzi G.; Russell A. E.; Fermin D. J. AMnO_3_ (A= Sr, La, Ca, Y) Perovskite Oxides as Oxygen Reduction Electrocatalysts. Top. Catal. 2018, 61 (3–4), 154–161. 10.1007/s11244-018-0886-5. 30956502 PMC6413806

[ref35] Jampaiah D.; Velisoju V. K.; Venkataswamy P.; Coyle V. E.; Nafady A.; Reddy B. M.; Bhargava S. K. Nanowire Morphology of Mono-and Bidoped α-MnO_2_ Catalysts for Remarkable Enhancement in Soot Oxidation. ACS Appl. Mater. Interfaces 2017, 9 (38), 32652–32666. 10.1021/acsami.7b07656. 28862428

[ref36] Yamaguchi Y.; Aono R.; Hayashi E.; Kamata K.; Hara M. Template-Free Synthesis of Mesoporous β-MnO_2_ Nanoparticles: Structure, Formation Mechanism, and Catalytic Properties. ACS Appl. Mater. Interfaces 2020, 12 (32), 36004–36013. 10.1021/acsami.0c08043. 32805787

[ref37] Borca C. N.; Canulescu S.; Loviat F.; Lippert T.; Grolimund D.; Döbeli M.; Wambach J.; Wokaun A. Analysis of the Electronic Configuration of the Pulsed Laser Deposited La_0. 7_Ca_0. 3_MnO_3_ Thin Films. Appl. Surf. Sci. 2007, 254 (4), 1352–1355. 10.1016/j.apsusc.2007.09.049.

[ref38] Yang Y.; Su X.; Zhang L.; Kerns P.; Achola L.; Hayes V.; Quardokus R.; Suib S. L.; He J. Intercalating MnO2 Nanosheets with Transition Metal Cations to Enhance Oxygen Evolution. ChemCatChem. 2019, 11 (6), 1689–1700. 10.1002/cctc.201802019.

[ref39] Celorrio V.; Calvillo L.; Dann E.; Granozzi G.; Aguadero A.; Kramer D.; Russell A. E.; Fermín D. J. Oxygen Reduction Reaction at LaxCa_1–x_MnO_3_ Nanostructures: Interplay between A-Site Segregation and B-Site Valency. Catal. Sci. Technol. 2016, 6 (19), 7231–7238. 10.1039/C6CY01105E.

[ref40] Bradley K.; Giagloglou K.; Hayden B. E.; Jungius H.; Vian C. Reversible Perovskite Electrocatalysts for Oxygen Reduction/Oxygen Evolution. Chem. Sci. 2019, 10 (17), 4609–4617. 10.1039/C9SC00412B. 31123571 PMC6492633

[ref41] Su H.-Y.; Gorlin Y.; Man I. C.; Calle-Vallejo F.; Nørskov J. K.; Jaramillo T. F.; Rossmeisl J. Identifying Active Surface Phases for Metal Oxide Electrocatalysts: A Study of Manganese Oxide Bi-Functional Catalysts for Oxygen Reduction and Water Oxidation Catalysis. Phys. Chem. Chem. Phys. 2012, 14 (40), 14010–14022. 10.1039/c2cp40841d. 22990481

[ref42] Kéranguéven G.; Royer S.; Savinova E. Synthesis of Efficient Vulcan–LaMnO_3_ Perovskite Nanocomposite for the Oxygen Reduction Reaction. Electrochem. Commun. 2015, 50, 28–31. 10.1016/j.elecom.2014.10.019.

[ref43] Kuznetsov D. A.; Han B.; Yu Y.; Rao R. R.; Hwang J.; Román-Leshkov Y.; Shao-Horn Y. Tuning Redox Transitions via Inductive Effect in Metal Oxides and Complexes, and Implications in Oxygen Electrocatalysis. Joule 2018, 2 (2), 225–244. 10.1016/j.joule.2017.11.014.

[ref44] Wang L.; Adiga P.; Zhao J.; Samarakoon W. S.; Stoerzinger K. A.; Spurgeon S. R.; Matthews B. E.; Bowden M. E.; Sushko P. V.; Kaspar T. C.; et al. Understanding the Electronic Structure Evolution of Epitaxial LaNi_1–x_Fe_x_O_3_ Thin Films for Water Oxidation. Nano Lett. 2021, 21 (19), 8324–8331. 10.1021/acs.nanolett.1c02901. 34546060

[ref45] Lin C.; Li J.-L.; Li X.; Yang S.; Luo W.; Zhang Y.; Kim S.-H.; Kim D.-H.; Shinde S. S.; Li Y.-F.; et al. In-situ reconstructed Ru atom array on α-MnO2 with enhanced performance for acidic water oxidation. Nat. Catal 2021, 4 (12), 1012–1023. 10.1038/s41929-021-00703-0.

[ref46] Lübke M.; Sumboja A.; McCafferty L.; Armer C. F.; Handoko A. D.; Du Y.; McColl K.; Cora F.; Brett D.; Liu Z.; et al. Transition-Metal-Doped α-MnO2 Nanorods as Bifunctional Catalysts for Efficient Oxygen Reduction and Evolution Reactions. Chemistryselect 2018, 3 (9), 2613–2622. 10.1002/slct.201702514.

[ref47] Celorrio V.; Leach A. S.; Huang H.; Hayama S.; Freeman A.; Inwood D. W.; Fermin D. J.; Russell A. E. Relationship between Mn Oxidation State Changes and Oxygen Reduction Activity in (La,Ca)MnO3 as Probed by In Situ XAS and XES. ACS Catal. 2021, 11 (11), 6431–6439. 10.1021/acscatal.1c00997.

