# Supplementary material for: Activating Mn Sites by Ni Replacement in α-MnO2
Source: ACS Mater Au. 2023 Nov 20;4(1):74–81. doi: 10.1021/acsmaterialsau.3c00051 (PMC10786130; doi:10.1021/acsmaterialsau.3c00051)
Supplement: Supplementary file 1 — mg3c00051_si_001.pdf [file mg3c00051_si_001.pdf]

Supporting Information

Activating Mn Sites by Ni Replacement in α-MnO_2

Sami M. Alharbi,[a,b] Mohammed A. Alkhalifah,[a,c] Benjamin Howchen,[a] Athi N. A. Rahmah[a], Veronica Celorrio,[d] and David J. Fermin[a]*

[a]School of Chemistry, University of Bristol, Cantocks Close, Bristol BS8 1TS, UK

[b]Department of Chemistry, College of Science, Qassim University, Buraydah 52571, Saudi Arabia

[c]Department of Chemistry, College of Science, King Faisal University, P.O. Box 380, Al-Ahsa, 31982, Saudi Arabia

[d]Diamond Light Source Ltd., Diamond House, Harwell Campus, Didcot OX11 0DE, UK

Figure S1. High Resolution TEM images of α-MnO_2 with various Ni content.

Figure S2. TEM-EDX images of α-MnO_2 with various Ni content.

Figure S3. XPS survey spectra of 5% and 25% Ni_{pre} samples.

Figure S4. The k^2-weighted EXAFS signals in *k*-space and magnitude of FT signal of the k^2-weighted EXAFS.

Figure S5. Koutecky-Levich plots of the ORR reaction at 0.70 V vs RHE at α-MnO_2 electrocatalysts with various Ni content.

Figure S6. LSV curves in the OER region including and excluding IR compensation as well as Tafel plots for the various Ni modified α-MnO_2.

Table S1. Elemental composition of α-MnO_2 with various Ni content as obtained from ICP-OES.

Table S2. Elemental composition of α-MnO_2 with various Ni content as obtained from EDX.

Table S3. Structural parameters obtained from EXAFS analysis.

Table S4. Effective number of electrons (*n*) and kinetically limited current (i_k) at 0.70 V vs RHE.

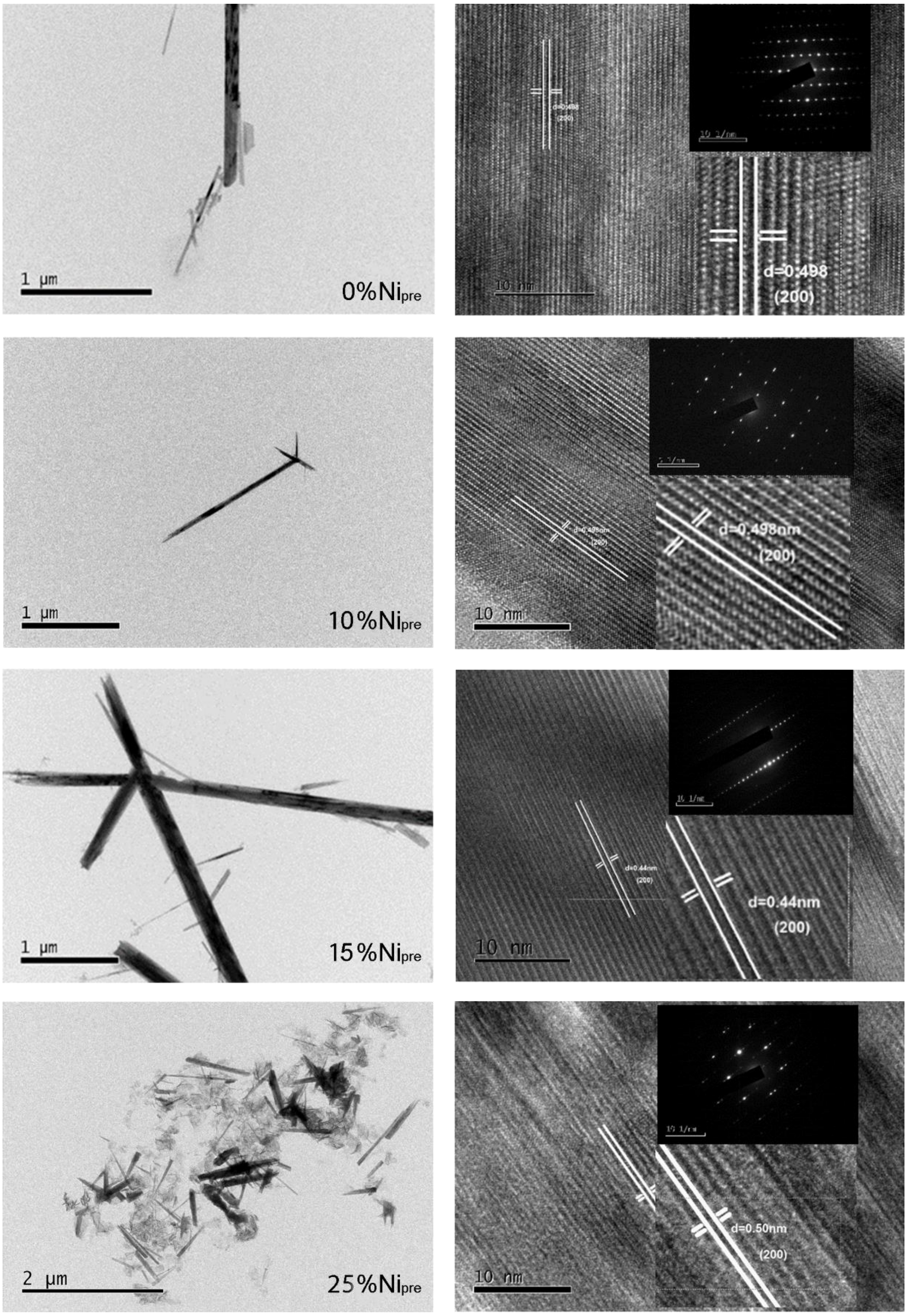

Figure S1. High Resolution TEM images of α-MnO_2 with various Ni content. α-MnO_2 were generated by hydrothermal growth with various Ni molar ratios in the precursor solution (Ni_{pre}). The images show the characteristic high aspect ratio of the hollandite phase and lattice fringes associated with the (200) planes. Featureless particles in 25% Ni_{pre} suggests segregation of a separate phase.

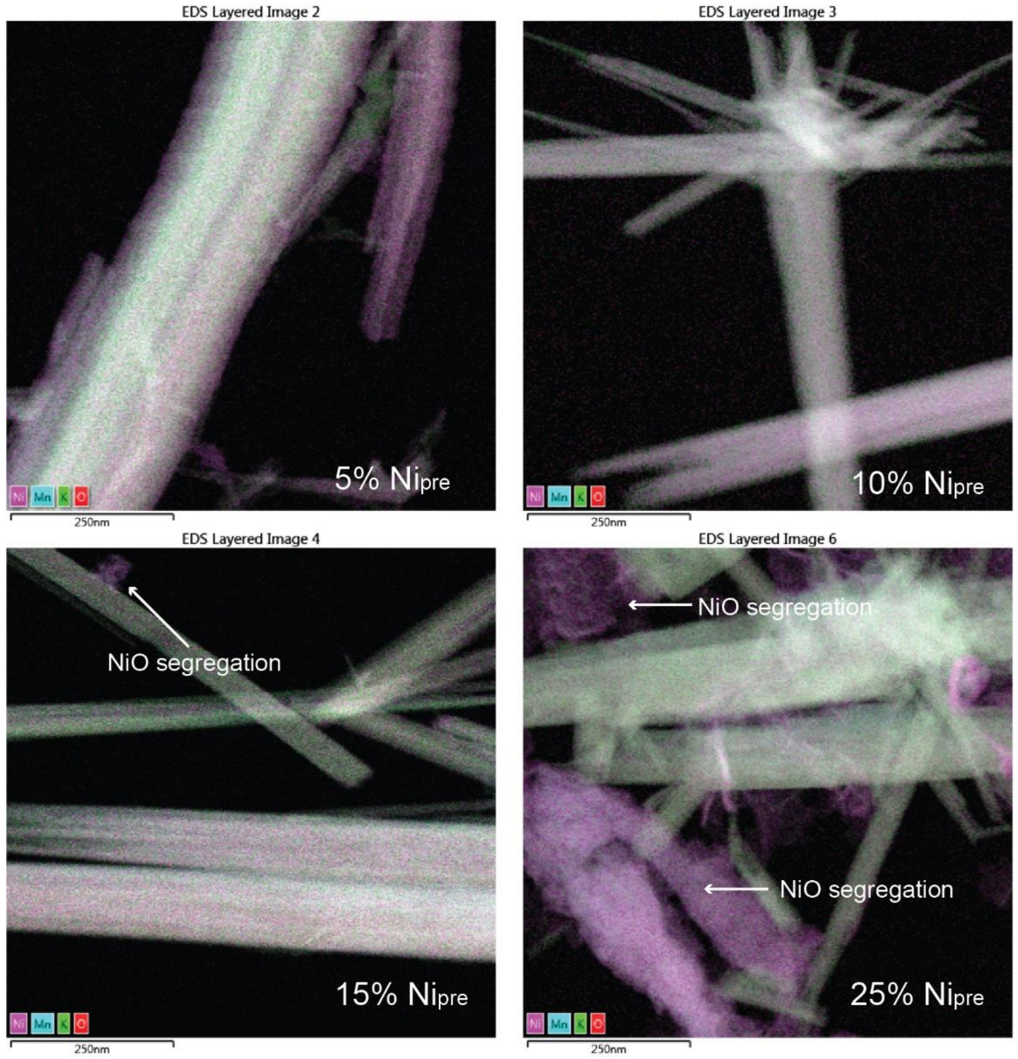

Figure S2. TEM-EDX images of 5 ,10 ,15 and 25% Ni_{pre} nanostructures. Images show Ni (pink) primarily located in the α-MnO_2 lattice in the case up to 15% Ni_{pre}. At 25% Ni_{pre} substantial segregation of Ni phases can be observed.

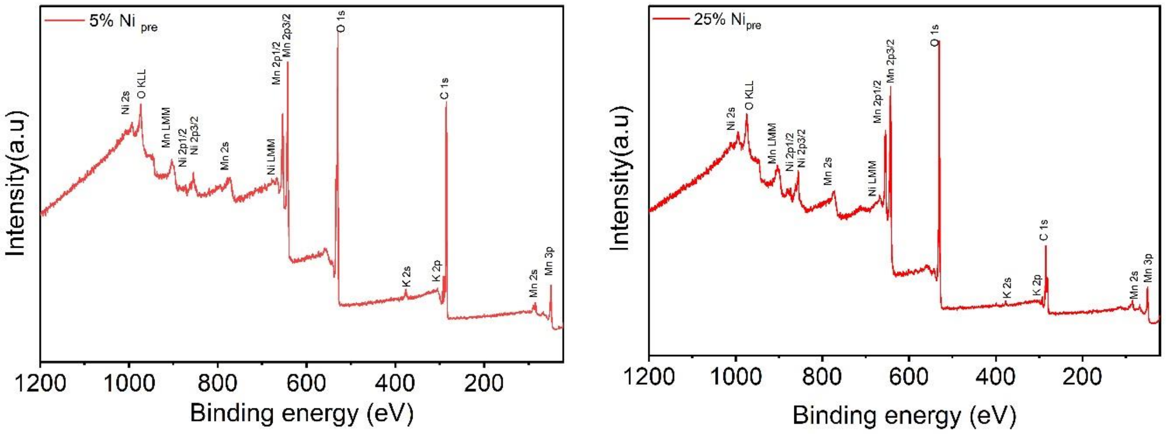

Figure S3. Survey XPS spectra of 5% Ni_{pre} (left) and 25% Ni_{pre} (right) samples.

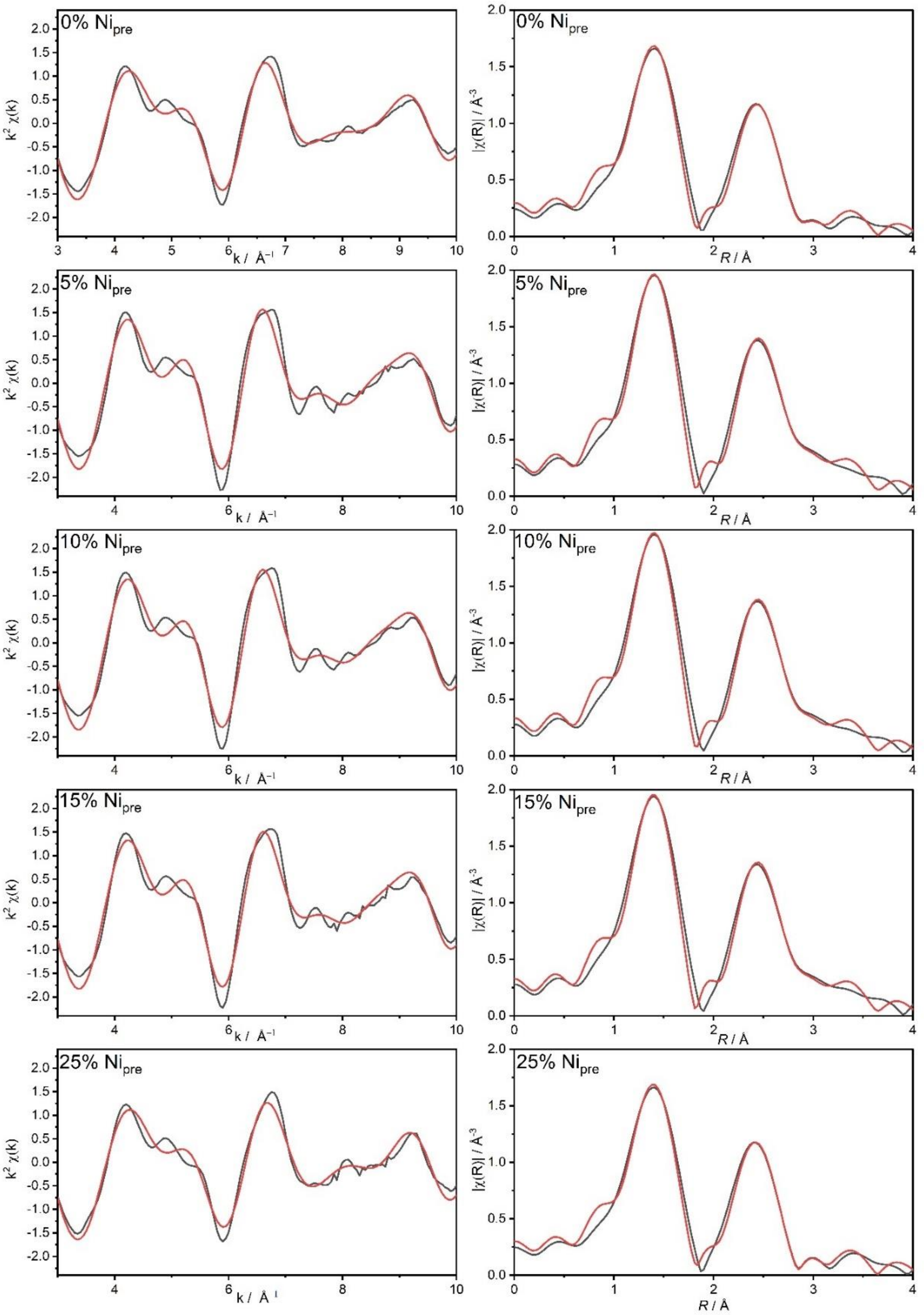

Figure S4 (Left) Data (red line) and fits (black line) of the k^2-weighted EXAFS signals in *k*-space of samples powder pellets. (Right) Data (red lines) and fits (black lines) of the magnitude of FT signal of the k^2-weighted EXAFS.

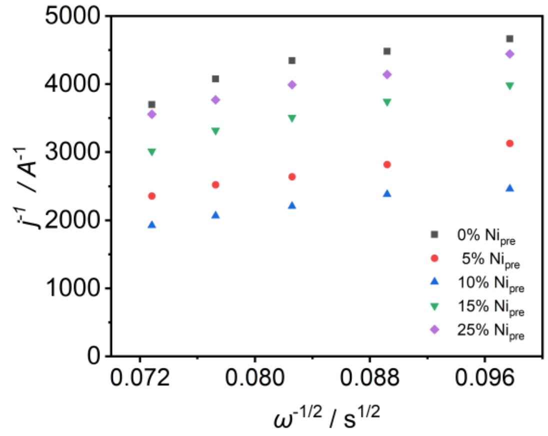

Figure S5. Koutecky-Levich plots of the ORR reaction at 0.70 V vs RHE at α-MnO_2 electrocatalysts with various Ni content. The catalyst loading at the electrode was 398 μg cm^{-2}. Measurements were conducted under O_2-saturated 0.1 M KOH solution at 0.70 V.

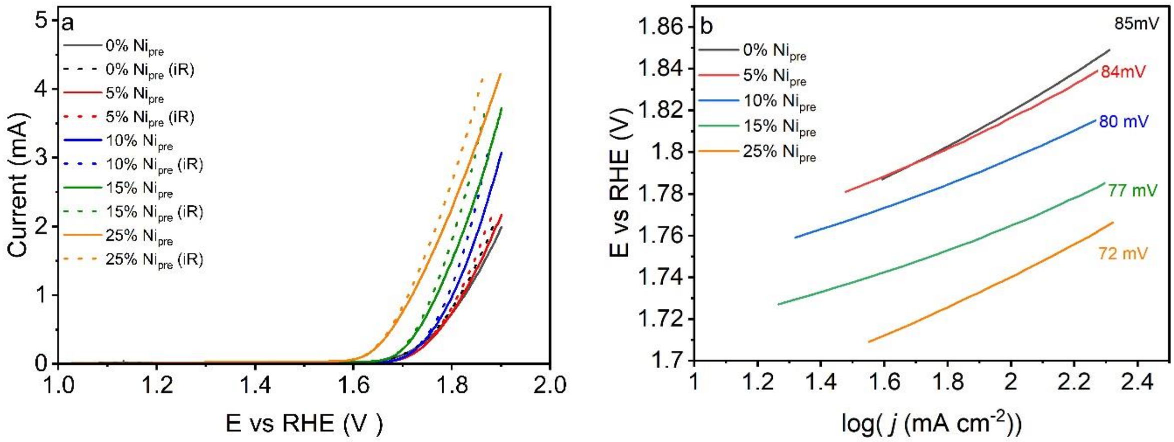

Figure S6. LSV curves in the OER region including and excluding IR compensation (a). Uncompensated resistance measured from electrochemical impedance spectroscopy is independent of the catalyst's composition with a mean value of 10±2 Ω. Tafel plots constructed from the LSV curves of the various Ni modified α-MnO_2.

Table S1. Elemental composition of α-MnO_2 with various Ni content as obtained from ICP-OES.

Precursor Ni/(Mn+Ni)	K (ppm)	Mn (ppm)	Ni (ppm)	Mean particle (Ni/Ni+Mn)
**0%Ni pre**	3.86	24.54	0	0
**5%Ni pre**	1.64	15.26	0.68	0.040
**10%Ni pre**	2.35	25.51	1.28	0.045
**15%Ni pre**	2.30	25.08	1.67	0.058
**25%Ni pre**	0.69	19.72	2.96	0.123

Table S2. Elemental composition of α-MnO_2 with various Ni content as obtained from EDX.

Precursor Ni/(Mn+Ni)	K (at%)	Mn (at%)	Ni (at%)	Mean particle (Ni/Ni+Mn)
**0%Ni $_{pre}$**	16.67	83.33	0.00	0.00
**5%Ni $_{pre}$**	13.86	82.67	3.47	0.040
**10%Ni $_{pre}$**	11.66	85.20	3.14	0.036
**15%Ni $_{pre}$**	10.94	83.77	5.28	0.059
**25%Ni $_{pre}$**	6.75	85.28	7.98	0.086

Table S3. Structural parameters obtained from EXAFS analysis. Relative energy shift and the best fit results from the structural analysis of the synthetized samples at the Mn K-edge. N is the coordination number; R is the interatomic distance and σ^2 is the Debye-Waller factor. R_f is the R-factor, which represents the relative error of the fit and data. Fitting range 2.8 < k <10.1 Å^{-1}, 1.0 < R < 3.8 Å.

	Shell	N	R / Å	σ^2 x 10^3 / Å^2	ΔE_0 / eV	R_f
α-MnO_2 (S_0^2 = 0.57 ± 0.07)	Mn-O_1	6	1.89 ± 0.02	2.6 ± 1.8	-1.3 ± 1.8	0.012
	Mn-Mn_1	4	2.88 ± 0.02	3.6 ± 1.9		
	Mn-Mn_2	4	3.43 ± 0.06	7.2 ± 5.3		
	Mn-O_2	7	3.53 ± 0.15	7.2 ± 5.3		
5% Ni_{pre} (S_0^2 = 0.59 ± 0.07)	Mn-O_1	6	1.89 ± 0.02	1.7 ± 1.6	-1.6 ± 1.8	0.015
	Mn-Mn_1/Ni_1	4	2.89 ± 0.02	2.9 ± 2.0		
	Mn-Mn_2/Ni_2	4	3.42 ± 0.04	5.7 ± 3.8		
	Mn-O_2	7	3.46 ± 0.14	5.7 ± 3.8		
10% Ni_{pre} (S_0^2 = 0.61 ± 0.08)	Mn-O_1	6	1.89 ± 0.02	1.7 ± 1.7	-1.6 ± 1.8	0.015
	Mn-Mn_1	4	2.89 ± 0.02	3.2 ± 1.7		
	Mn-Mn_2	4	3.42 ± 0.03	6.1 ± 3.9		
	Mn-O_2	7	3.47 ± 0.14	6.1 ± 3.9		
15% Ni_{pre} (S_0^2 = 0.60 ± 0.07)	Mn-O_1	6	1.88 ± 0.02	1.7 ± 1.7	-1.8 ± 1.7	0.015
	Mn-Mn_1/Ni_1	4	2.88 ± 0.02	3.3 ± 2.0		
	Mn-Mn_2/Ni_2	4	3.42 ± 0.04	6.0 ± 4.4		
	Mn-O_2	7	3.46 ± 0.13	6.0 ± 4.4		
25% Ni_{pre} (S_0^2 = 0.57 ± 0.06)	Mn-O_1	6	1.89 ± 0.02	2.9 ± 1.6	-1.2 ± 1.4	0.011
	Mn-Mn_1	4	2.87 ± 0.02	3.5 ± 1.5		
	Mn-Mn_2	4	3.43 ± 0.06	6.7 ± 6.5		
	Mn-O_2	7	3.54 ± 0.12	6.7 ± 6.5		

Table S4. Effective number of electrons (*n*) and kinetically limited current (i_k) estimated at 0.70 V vs RHE obtained from the rotating ring-disk electrode responses at various angular rotation rates.

Precursor Ni/(Mn+Ni) %	***n***	**$-i_K \times 10^{-3}$(A)**
**0**	2.34 ±0.17	0.82±0.01
**5**	3.78 ±0.19	3.25±0.45
**10**	4.00 ±0.12	5.05±0.45
**15**	3.00 ±0.11	2.65±0.05
**25**	2.47 ±0.10	0.96±0.04
